# Mr. Grose, on the Use of Yeast and Cold Affusion in Typhus

**Published:** 1800-11

**Authors:** I. H. Grose

**Affiliations:** Winslow


### *To the Editors of the Medical and Phyſical Journal.*

Gentlemen,

It affords me the greateſt ſatisfaction, that I have it in my power to corroborate the teſtimony of a reſpectable practitioner, relative to the highly antiputreſcent properties of the flos cerivifiæ, which in no caſe has appeared more conſpicuous than in the following:—

The ſon of W. M. of this town, ætat. 14, was attacked on the 10th of July, 1800, with the uſual ſymptoms of Typhus, attended with violent pain in the head, and a fixed pain in the abdomen. Judging the caſe to be highly dangerous, I ordered him a ſtrong decoction of bark, with powder, &c. and aromatics; a free uſe of Port wine; and directed that every thing he drank ſhould be made ſour with with the acidum vitriolicum aromaticum. 11th, Still continues exceeding ill; pulſe quick and ſmall; applied the thermometer, which immediately roſe to 100; ſkin very dry and the mouth and fauces furred and black. I ſaw him again the ſame evening, when he was highly delirious, nor had the quantity of bark he had taken any effect in counteracting the putrid diatheſis: the pain in the ſtomach ſtill continued; and the fæces were regularly diſcharged, but quite black and offenſive. Applicet. emplaſt. canth. dorſo. 12th, The fever ſtill continues with little alteration; he is quite comatoſe this morning; no tendency to perſpiration, but ſkin quite dry. Having peruſed an account in your excellent Journal, of the utility of bathing the patient during the hot fit, I determined to give it a trial, and alſo to lay aſide the bark; and as the caſe appeared to me a loſt one, rely wholly on this method and a liberal uſe of yeaſt. The very idea of applying cold water during the febrile heat, appears to an unenlightened mind very dangerous; and the father of the patient being a poor man, and uninformed, I found ſome difficulty in procuring his conſent; neverthelefs, having repreſented to him the imminent danger of the caſe, and aſſuring him it had been repeatedly practiſed with ſucceſs, he acquieſced in my advice, and determined to put it in immediate execution. The method recommended by Dr. George Moſſman, I conceived to be the beſt, and accordingly I ſponged him all over with cold vinegar, and directed the free uſe of yeaſt and wine. He was ſponged again at night, and I ſaw him ſoon afterwards evidently better, his pulſe being regular and body cool: He had a good night's reſt; and on applying the thermometer next morning, it was at 80. Finding ſuch a beneficial effect, and in ſo ſhort a time, I deſired that the yeaſt (the doſe of which was a large ſpoonful

ſpoonful ſix or eight times a day) and the uſe of the vinegar might by no means be omitted. From this period he became evidently better; in two days after the yeaſt was diſcontinued: the quantity he had taken was about a pint, and he had been ſponged four times.

On conſideration of the wonderful change which ſo rapidly ſucceeded the adminiſtration of the yeaſt, I cannot but imagine it to be the beſt remedy in putrid fevers ever yet diſcovered. It is well known to ſea-faring men, that if meat, after having become putrid, is immerſed in yeaſt, or only covered with it for the ſpace of thirty-ſix hours, it becomes as freſh and as good as ever. This intelligence I had from an officer in the navy, who had tried it many times. It may be urged in reply, that however potent a medicine may be when applied externally, it has not the ſame effect internally taken; this has been proved in the analyſis of lithontriptic remedies. Have not mercurial preparations the ſame efficacy (or nearly) in cauſing a ptyaliſm, whether externally applied or internally adminiſtered? Why is relief expected from the emplaſtrum aſafœtidæ, but that the antiſpaſmodic properties of the aſafœtida are taken by the abſorbents into the ſyſtem?

With reſpect to the efficacy of cold bathing, (or ſponging, which is nearly ſimilar) it is too well known to need any comment; nor are its valuable properties by any means confined to one particular complaint. I have witneſſed its good effects in phthiſis, when every other remedy had failed; by bathing the cheſt morning and evening with cold water, it has leſſened the frequency of the cough, kept the tongue moiſt, and the patient has experienced from its uſe the moſt grateful ſenſations. Perhaps I may be too ſanguine in my expectations, I may too fondly cheriſh the idea; but it is ſtill impreſſed on my mind, that if the generality of medical practitioners would give it a few trials, it will be found a moſt valuable remedy. It is not from one or two inſtances that I wiſh any one to form even an opinion, for certainly no deciſion can be made: But particularly among the lower claſſes of people in country villages, where medical aid cannot be immediately obtained, it would be an act of charity if the clergyman, or any conſiderate inhabitant, would ſee it duly adminiſtered; and I am confident they would reap that heart-felt ſatisfaction which is ever the attendant on humanity. I have the honour to be,

GENTLEMEN,

Your humble ſervant,

*Winſlow, July* 26, 1800. I. H. GROSE.

P. S. It has been urged againſt the uſe of yeaſt, that it is violently purgative; this is what I never ſaw, but, on the contrary, it had always the effect of a cordial.

